# Addressee Identity and Morphosyntactic Processing in Basque Allocutive Agreement

**DOI:** 10.3389/fpsyg.2017.01439

**Published:** 2017-09-05

**Authors:** Max Wolpert, Simona Mancini, Sendy Caffarra

**Affiliations:** ^1^Basque Center on Cognition, Brain and Language San Sebastian, Spain; ^2^Integrated Program in Neuroscience, McGill University Montreal, QC, Canada; ^3^Centre for Research on Brain, Language and Music Montreal, QC, Canada

**Keywords:** addressee, interlocutor, Basque, allocutive, morphosyntax, pragmatics

## Abstract

Information about interlocutor identity is pragmatic in nature and has traditionally been distinguished from explicitly coded linguistic information, including mophosyntax. Study of speaker identity in language processing has questioned this distinction, but addressee identity has been less considered. We used Basque to explore how addressee identity is processed during morphosyntactic analysis. In the familiar register *hika*, Basque has obligatory allocutive agreement, where verbal morphology represents the gender of a non-argument addressee. We manipulated the gender of the allocutive verb and the congruence of addressee gender in conversations between two interlocutors. Items with person agreement manipulations were included as a control comparison. Basque speakers familiar with *hika* completed speeded acceptability judgments and unspeeded, offline naturalness ratings for each conversation. Results showed a main effect of addressee identity congruence for naturalness ratings, but there was no main effect for addressee identity congruence for reaction times or accuracy in the acceptability judgment. Interactions and correlations with biographical data showed that the effect of congruence was modulated by the gender of the allocutive verb and that *hika* proficiency was related to participants' performance for the acceptability judgment. These results show an interaction between morphosyntactic and pragmatic information and are the first experimental data of allocutive processing. In comparison, clear effects were seen for the person agreement condition, indicating that person disagreement is more disruptive to processing than addressee identity incongruence. This study has implications for investigation of the role of extralinguistic information in morphosyntactic processing, and suggests that not all such information plays an equal role.

## Introduction

Social context is essential for complete language comprehension, and listeners must consider this information online during speech processing. Interlocutor identity is one example: saying ‘I love you’ to your mother is not the same as saying it to your boss, and the meaning is not the same if the response is ‘I love you, too.’ The distinction between romantic and familial love in this case is pragmatic, or extralinguistic, in nature, relying on listeners' implicit social knowledge about speakers' intended meaning for specific interlocutors. This contrasts with morphosyntactic information, which is explicitly present in speech. For example, to say “she is tired” in Spanish, one says *ella está* ***cansada*** and not ^*^ella está ***cansado***, with agreement between the adjective and the gender of the subject in the final vowel of ***cansada***. Explicit linguistic meaning, like that from morphosyntax, has traditionally been distinguished from pragmatic meaning (e.g., Grice, 1975), but the boundary is not always clear. Such is the case with Basque allocutive agreement, where context information, the gender of the addressee, is coded for morphosyntactically, even though the addressee is not an argument of the verb. In this study, we report behavioral effects of manipulating the congruence of interlocutor identity and allocutive verb forms, which allowed investigation of the role of addressee identity in morphosyntactic processing.

Interlocutor identity has mostly been studied from the perspective of the speaker. This includes evidence from eye-tracking about shared semantic knowledge (Hanna et al., 2003; Metzing and Brennan, 2003; for review see Barr and Keysar, 2006) and speaker reliability (Grodner and Sedivy, 2011), which showed that speaker identity affected which objects participants looked at first. Event-related potential (ERP) experiments have further shown that semantic processing is affected by the perception of a speaker's ability to accomplish an action (Bornkessel-Schlesewsky et al., 2013) and social stereotypes (Lattner and Friederici, 2003; Van Berkum et al., 2008), where, for example, a male voice talking about applying makeup elicits a greater early negativity than a female voice. Speaker ERP effects extend to morphosyntactic processing as well, including foreign accented speech removing the ERP effects from gender errors (Hanulíková et al., 2012) and an increased early negativity for incongruence between speaker gender and subject-verb gender agreement (Hanulíková and Carreiras, 2015). Some behavioral evidence showing slower reaction times (RTs) in lexical decision tasks with bilingual interlocutors (Molnar et al., 2015; Martin et al., 2016) also supports the idea that speaker identity is integrated early and automatically, and other work has shown slower RTs when grammatical gender does not match speaker gender (Andonova, 2013; Vitevitch et al., 2013).

Less is known about the processing of addressee identity. In one study, researchers compared the processing of addressee-directed and overheard responses to a mock job interview and reported similar functional magnetic resonance imaging (fMRI) activation patterns (Bašnáková et al., 2015). More relevant to the current investigation, studies investigating politeness (Jiang et al., 2013; Jiang and Zhou, 2015a,b) showed an early and disambiguating ERP effect of incongruence between interlocutor social status and formal or informal pronouns. Research has also been done on the side of morphosyntax with Japanese honorifics, which show interlocutor social status in verbal morphology. In a behavioral study (Yoshimura and MacWhinney, 2010), participants relied on honorific cues for interpretation in the absence of overt subjects and case markers, but this processing was about 100 ms slower than that for overt case markers. In another study of honorifics using fMRI (Momo et al., 2008), researchers compared violations in four conditions—honorification, morphosyntactic, semantic, and spelling—and reported similar neural correlates for processing of honorifics and morphosyntax. A behavioral task was also included and showed that both the honorification and morphosyntactic conditions had lower accuracy and longer RTs than the semantic and spelling conditions. However, these studies did not manipulate the congruence between honorification and addressee, so the results may not represent processing of extralinguistic information. All the studies mentioned so far using neuropragmatics methods give valuable information about the neural correlates and time course of processing, but more behavioral evidence is needed to demonstrate the robustness of interlocutor identity effects.

With relatively little work addressing the issue, it is still unclear how addressee identity interacts with sentence processing. This leads to the present study, which manipulated morphosyntactic congruence with addressee gender in Basque. Basque has two second-person singular registers, which will be referred to here as *zuka* and *hika*. *Zuka* is the standard register used for most interaction, while *hika* is a familiar register indicating a high degree of closeness between interlocutors. Treatment in *hika* has obligatory allocutive agreement, where inflection of the auxiliary verb agrees with the non-argument addressee (Oyharçabal, 1993; Hualde and de Urbina, 2003; Antonov, 2015). Basque allocutive agreement has two variations for addressee gender; while the verbal morphology is complex, the masculine verb form generally includes *-k/-a-* and the feminine verb form *-n/-na-*. Besides allocutive agreement, Basque has no grammatical marking for gender and no gender-marked pronouns. Examples of treatment in both *zuka* and *hika*, with masculine and feminine allocutive verb forms underlined, are given below.

(i)   *Astelehenak   gogorrak   izaten   dira     beti*.          (**zuka)**
        Mondays        hard           are        AUX   always
        ‘Mondays are always hard.’
(ii)*  Astelehenak   gogorrak   izaten* *dituk*                         *beti*.          (**hika**)
        Mondays        hard           are        AUX.ALLO_MASC_     always
        ‘Mondays are always hard.’ [male addressee]
(iii)  *Astelehenak  gogorrak   izaten* *ditun*                     *beti*.        (**hika**)
        Mondays        hard           are    AUX.ALLO_FEM_       always
        ‘Mondays are always hard.’ [female addressee]
        AUX = auxiliary verb, ALLO = allocutive verb,
        MASC = masculine, FEM = feminine

Because no experimental work on processing has been done with allocutive agreement, it is unclear from a psycholinguistic perspective how agreement with a non-argument is different from other better-studied forms of agreement. Person, number and gender are all potential cues for resolving agreement dependencies (Corbett, 1983), and person agreement is an ideal comparison for allocutive agreement. Both person and allocutive agreement have a referent reflected in verbal morphology, and the relevance of the comparison is emphasized by the fact that, similar to allocutive, person interpretation involves the analysis of discourse information (Benveniste, 1966; Sigurdsson, 2004; Mancini et al., 2013) to draw a link between morphosyntax and the discourse roles that a subject argument bears. Processing effects of person agreement are already documented in behavioral tasks with acceptability judgments, and judgments for person disagreement have been found to have faster RTs than person agreement (Mancini et al., 2011a,b; Perez et al., 2012; Zawiszewski et al., 2016; but see Mancini et al., 2014 for different results in a self-paced reading study). This indicates that behavioral measures can clearly capture the detection of person violations and identifying a person error is easier than identifying the absence of one. The present study explored the processing of allocutive agreement in Basque speakers with person agreement as a well-established control comparison. Any behavioral differences between allocutive and person agreement performance can reveal the nature of the interaction between morphosyntax and pragmatics.

In the context of this study, there are several important points to keep in mind about Basque allocutivity. First, the dialect discussed in the present study is the standard dialect Batua, which is the form taught in schools (Hualde and Zuazo, 2007), but the Basque language consists of multiple dialects, and *hika* usage and morphology have variation among communities (Alberdi, 1994; Elordieta et al., 1999; Lizardi and Munduate, 2015). Second, *hika* treatment is traditionally socially restricted, most used by and between men and older speakers, and many Basque speakers do not know how to use the complicated verbal morphology required for allocutive agreement (Echeverria, 2001, 2003, 2010; Haddican, 2003, 2005, 2007). Male speakers may also occasionally address female interlocutors with masculine verb forms (Echeverria, 2003). Third, although allocutivity has been most studied in Basque, it is not unique to the language. Extensive verbal morphology changes related to addressee identity also occur with Japanese and Korean honorifics, and several other languages also change verbal morphology to agree with non-argument addressee gender (Antonov, 2013, 2015).

Here we investigated how addressee gender affects the processing of Basque allocutive agreement. Conversations in *hika* were created with two manipulated factors: Congruence (addressee gender congruent or incongruent with allocutive verb form) and Allocutive (masculine or feminine allocutive verb form). Basque morphosyntactic processing has previously been investigated experimentally, including person and number agreement, word order, and ambiguity resolution (Erdocia et al., 2009; Zawiszewski and Friederici, 2009; Santesteban et al., 2013; Zawiszewski et al., 2016); however, to our knowledge, this study is the first experimental investigation of allocutivity. Additionally, the ERP and fMRI work summarized above has given an index of pragmatic processing, which is only indirectly linkable to behavior, while here behavior was tested directly.

To measure the effects of Allocutive and Congruence, two tasks were created for each conversation: an acceptability judgment, which was a time-constrained yes-no response, and a naturalness rating, a seven-point scale response without time pressure. The tasks differed on two dimensions: first, the type of question; second, the time constraint. Regarding the first difference, the two tasks could monitor qualitatively different aspects of language comprehension. While the acceptability judgment task focuses on the type of constructions that violate grammatical rules, the naturalness rating reflects what forms participants actually use. The use of a construction does not always imply grammatical acceptance and vice versa (Greenbaum, 1976), so the use of both tasks allows distinction of cases where participants reject a conversation according to hard rules but still recognize it as natural on a gradient judgment (Sorace and Keller, 2005). Regarding the second difference, speeded and unspeeded responses can capture different stages of the linguistic computation (Lewis and Phillips, 2015); while speeded online judgments tap into early, automatic language comprehension, unspeeded responses more likely reflect metalinguistic processes of reanalysis (Marinis, 2010).

We predicted that if addressee identity has an impact on morphosyntactic processing of Basque allocutive agreement, then there would be a main effect of Congruence in both tasks. Additionally, because *hika* is used mostly between men and there have been reports that masculine allocutive forms are used for female addressees (Echeverria, 2003), we predicted that masculine incongruent forms would be more accepted than feminine incongruent forms, meaning lower accuracy for the acceptability judgment and higher naturalness ratings. Different results between the tasks would be related to a distinction between linguistic prescriptive competence and daily usage of *hika*, as well as to the temporal dynamics of allocutive processing.

As a comparison for the addressee identity effect, conversations with person manipulations were also included in the standard register *zuka*. The same tasks as described previously were used for the person manipulation items, and we predicted faster RTs and lower naturalness ratings for items with disagreement. We also intended to compare the allocutive and person agreement conditions to show processing distinctions between linguistic and extralinguistic information, even when both are explicitly present in morphosyntax.

## Materials and methods

### Participants

Thirty-four native Basque speakers participated in the experiment. Seven participants had accuracy below the inclusion criterion of two standard deviations from the mean for either person or allocutive conditions and their data were not included for analysis. The 27 participants (13 female) included for analysis ranged in age from 23 to 50 years, with a mean age of 31.5 years (*SD* = 7.1), and were all healthy, right-handed, and familiar with *hika* (mean *hika* comprehension level 96 on a scale from 0 to 100, *SD* = 6). Participants were paid for participation and gave written informed consent.

### Materials

For the allocutive manipulation, 160 two-utterance Batua conversations in *hika*, each with four versions, were prepared between two speakers, Speaker A and Speaker B. Speaker A preceded Speaker B in each conversation. Two native Basque speakers, male and female, recorded Speaker A's sentences. Each speaker recorded half of the sentences individually and the other half repeating after the other speaker's recording to minimize differences in pacing and prosody. The durations of the sentences were similar between the male and the female speaker [female: mean = 3620 ms, *SD* = 672 ms; male: mean = 3655 ms, *SD* = 690 ms, *t*_(318)_ = 0.47, *p* = 0.64]. Another male native Basque speaker recorded Speaker B's sentences once with a masculine and once with a feminine allocutive verb form. The masculine and feminine forms of each sentence were recorded consecutively, with the feminine form recorded first for half and second for the other half, to minimize differences in pacing and prosody. The target allocutive verb always appeared as the final word in Speaker B's utterance and the masculine and feminine forms had a similar duration [feminine: mean = 551 ms, *SD* = 135 ms; masculine: mean = 547 ms, *SD* = 146 ms, *t*_(318)_ = 0.20, *p* = 0.84] and frequency [feminine: mean = 64, *SD* = 219; masculine: mean = 58, *SD* = 118, *t*_(318)_ = 0.30, *p* = 0.76] according to the Euskal Hiztegiaren Maiztasun Egitura database (Acha et al., 2014). Audio files were combined to form four versions of each conversation, as shown in Table 1A. The same Speaker B utterance was preceded by the male and female Speaker A utterances with a gap of 500 ms between Speaker A's offset and Speaker B's onset.

**Table 1:** Experimental conditions for allocutive items **(A)** and person items **(B)** with example dialogues.

**(A)**				
	**Congruent addressee gender**		**Incongruent addressee gender**	
Feminine allocutive	Female Speaker A:	*Astelehenak gogorrak izaten dituk beti*	Male Speaker A:	*Astelehenak gogorrak izaten dituk beti*
		Mondays are always hard		Mondays are always hard
	Speaker B:	*Asteko egunik gogorrenak horiek izaten **ditun***	Speaker B:	^*^Asteko egunik gogorrenak horiek izaten **ditun**
		They are the hardest day of the week		They are the hardest day of the week
Masculine allocutive	Male Speaker A:	*Astelehenak gogorrak izaten dituk beti*	Female Speaker A:	*Astelehenak gogorrak izaten dituk beti*
		Mondays are always hard		Mondays are always hard
	Speaker B:	*Asteko egunik gogorrenak horiek izaten **dituk***	Speaker B:	^*^Asteko egunik gogorrenak horiek izaten **dituk**
		They are the hardest day of the week		They are the hardest day of the week
**(B)**				
	**Person agreement**		**Person disagreement**	
	Female Speaker A:	*Txoriek ez dute zailtasunik izaten jatekoa lortzeko*	Male Speaker A:	*Txoriek ez dute zailtasunik izaten jatekoa lortzeko*
		Birds do not have any difficulty getting food		Birds do not have any difficulty getting food
	Speaker B:	*Egia, hango txori horrek ogi puska bat aurkitu **du***	Speaker B:	^*^Egia, hango txori horrek ogi puska bat aurkitu **dut**
		True, that bird has found a piece of bread		^*^True, that bird have found a piece of bread

*The English translation is provided below each sentence. The critical verb is bold and underlined. Ungrammatical sentences are preceded by an asterisk*.

An additional 60 two-utterance Batua conversations in *zuka*, each with two versions, were prepared, also between Speaker A and Speaker B. Half of Speaker A's sentences were recorded by the same female speaker and half by the same male speaker as the *hika* sentences. All of Speaker B's utterances had third person singular subjects and were recorded by the same male speaker as the *hika* sentences. Each of Speaker B's sentences was recorded once with person agreement and once with person disagreement (with a first-person singular subject verb form), and both versions of each sentence were recorded consecutively with the order counterbalanced. The target verb always appeared as the final word in Speaker B's utterance. Audio files were combined to form two versions of each conversation, as shown in Table 1B, with each of the two versions of Speaker B's sentences preceded by the same Speaker A's sentence.

For the allocutive manipulation, the four versions of each conversation were distributed in four different lists such that each condition was equally represented in each avoiding repetition of items. For the person manipulation, the two versions of each conversation were distributed in the previously created lists in the same manner with the male and female Speaker As equally represented in each list. In all, each list contained 220 items, 160 with allocutive manipulations and 60 with person manipulations, distributed randomly so that the person manipulation items were interleaved throughout the experiment. In each list, the following lexical variables were similar across all experimental conditions for Speaker B's utterances for the allocutive manipulation (all *p*s > 0.05): number of transitive and ditransitive verbs, present and past tense, and singular and plural subjects. Fourteen Basque speakers (11 female, mean age = 25, *SD* = 3.7) who did not participate in the study rated 20 recordings each from the male and female speakers for Speaker A as identifiable by speaker gender (five-point scale with 5 being extremely confident and 1 being not at all confident, female voice: mean confidence = 4.95, *SD* = 0.27; male voice: mean confidence = 4.95, *SD* = 0.24).

### Procedure

Participants wore headphones and sat in front of the experiment laptop in a quiet room. They were instructed to listen to conversations and make 1) acceptability judgments (“*Press ‘YES’ if the conversation was acceptable and press ‘NO’ if it was not acceptable.”)* and 2) naturalness ratings (“*Say how typical the conversation was from 1 (not typical at all) to 7 (very typical).”)* from one to seven. Participants were also verbally instructed to respond as quickly as possible for the acceptability judgment but not to worry about speed for the naturalness rating. The acceptability judgment was a yes-or-no response made by pressing the corresponding key on the keyboard, with the position of the yes-no buttons counterbalanced across participants. Response times were measured from the offset of Speaker B's utterance. Stimuli were presented from one of the four lists according to participant number.

Each trial began with the presentation of a fixation cross, which remained on the screen while the audio file played. The mean durations of the allocutive and person manipulation conversations were 8.5 and 8.3 s respectively. After the audio file, a question mark appeared on the screen during the acceptability judgment, followed by a 300 ms blank screen before the naturalness rating, where a scale from 1 to 7 was displayed with 1 being *batere ez ohikoa* (not at all typical) and 7 being *oso ohikoa* (very typical). The inter-stimulus interval was 1000 ms with a blank screen. We instructed participants to respond quickly for the acceptability judgments, and responses taking longer than 3 s were not recorded. There was no time restriction for naturalness ratings. Participants took a scheduled break after half the trials, received feedback on their accuracy and speed for the acceptability judgment, and resumed when they were ready. The experiment lasted around 45 min. Participants completed a questionnaire with biographical information, including age of acquisition (AoA), self-ratings of proficiency and comprehension, and language use for Spanish, Basque, and *hika*. Each participant was debriefed after completing the experiment and biographical questionnaire.

### Data analysis

The dependent variables evaluated were accuracy and reaction times (RTs) for the acceptability judgment and the scores (1–7) from the naturalness ratings. Only RTs from correct responses were considered and these were cleaned by excluding results two or more standard deviations away from the mean for the allocutive and person manipulations. The allocutive results were analyzed by subject and by item with a 2 × 2 repeated measures ANOVA with Allocutive verb form (masculine or feminine) and Congruence (congruent or incongruent) as within-subject and within-item factors. The person results were also analyzed by subject and by item with a one-way repeated measures ANOVA with Agreement (agreement or disagreement) as a within-subject and within-item factor. A significance level of 0.05 was used for all statistics. Significant interactions were analyzed using *post-hoc t*-tests with a False Discovery Rate correction to control for Type I errors (Benjamini and Hochberg, 1995). For experimental measures that showed significant main effects of or interactions with Congruence, a Pearson's product-moment coefficient was calculated for the effect of Congruence (congruent minus incongruent) and the *hika* behavioral measures from the biographical questionnaire (percent of time *hika* is used while speaking Basque, production score, and comprehension score). This was done as an exploratory step to see how self-reported *hika* experience related to processing of addressee identity^1^.

## Results

### Biographical questionnaire

Self-reported biographical data is summarized in Table 2. All participants were early Basque-Spanish bilinguals dominant in Basque, with average AoA for Basque and Spanish before one and before 6 years old respectively. Proficiency was high in both languages, and percent usage shows that participants were Basque dominant. Additionally, 85% of participants heard more masculine than feminine allocutive forms, 7% heard more feminine forms, and 7% heard both forms in equal proportions. All participants reported that *hika* use between two male speakers was most typical, and 37 and 56% reported that a man addressing a woman or a woman addressing a man in *hika* was least typical, respectively.

**Table 2:** Participant biographical data.

	**Mean ± *SD***
Age	31.5 ± 7.1
**BASQUE**	
AoA	0.2 ± 0.56
Speaking, 0–100	95.9 ± 4.7
Comprehension, 0–100	99.3 ± 2.3
Percent use	82.2 ± 17.4
Batua speaking, 0–100	87.0 ± 16.9
Batua comprehension, 0–100	98.7 ± 3.6
Percent of Batua use during week	33.7 ± 30.3
**HIKA**	
Age first exposure	1.6 ± 3.7
Age first use	10.1 ± 5.9
Speaking, 0–100	72.7 ± 19.2
Comprehension, 0–100	95.7 ± 5.5
Percent use when speaking Basque	40.0 ± 25.8
Percent of *hika* speakers in living area	46.5 ± 23.3
**SPANISH**	
AoA	5.7 ± 2.9
Speaking, 0–100	82.4 ± 11.7
Comprehension, 0–100	96.7 ± 6.7
Percent use	23.4 ± 21.0

### Allocutive results

**Figure 1:**
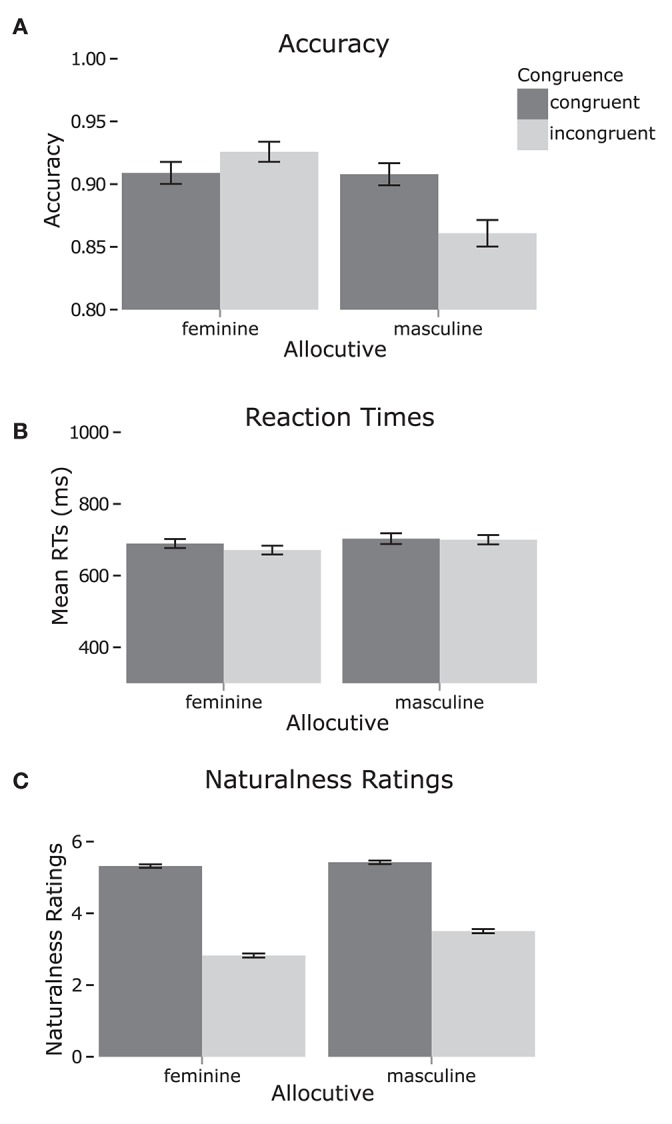
Results for the allocutive manipulation. **(A,B)** Show accuracy and RTs respectively for the acceptability judgments. **(C)** Shows naturalness ratings results. Error bars show standard error.

The ANOVA results are summarized in Table 3 and the data are shown in Figure 1. Additionally, Pearson's product-moment coefficients were calculated for subjects' results between tasks. Neither accuracy [*r* = 0.07, *p* = 0.6] nor RT [*r* = −0.03, *p* = 0.8] were correlated with naturalness ratings.

**Table 3:** ANOVA results for allocutive manipulation conditions with by subject (F1) and by item (F2) results.

**Accuracy**		
Congruence	*F1*_(1, 26)_ = 0.7, *p* = 0.4, η^2^ = 0.03	*F2*_(1, 159)_ = 0.1, *p* = 0.7, η^2^ < 0.01
Allocutive^*^	*F1*_(1, 26)_ = 7.4, *p* = 0.012, η^2^ = 0.22	*F2*_(1, 159)_ = 7.8, *p* = 0.006, η^2^ = 0.05
Congruence × Allocutive^*^	*F*1_(1, 26)_ = 4.5, *p* = 0.04, η^2^ = 0.15	*F2*_(1, 159)_ = 5.5, *p* = 0.02, η^2^ = 0.03
**RTs**		
Congruence	*F1*_(1, 26)_ = 1.9, *p* = 0.2, η^2^ < 0.01	*F2*_(1, 157)_ = 0.7, *p* = 0.39, η^2^ < 0.01
Allocutive	*F1*_(1, 26)_ = 0.05, *p* = 0.8, η^2^ = 0.07	*F2*_(1, 157)_ = 3.9, *p* = 0.05, η^2^ = 0.02
Congruence × Allocutive	*F*1_(1, 26)_ = 0.3, *p* = 0.6, η^2^ = 0.01	*F*2_(1, 157)_ = 1.4, *p* = 0.2, η^2^ < 0.01
**Naturalness Ratings**		
Congruence^***^	*F1*_(1, 26)_ = 52.6, *p* < 0.001, η^2^ = 0.67	*F*2_(1, 159)_ = 1786, *p* < 0.001, η^2^ = 0.92
Allocutive^**^	*F1*_(1, 26)_ = 11.8, *p* = 0.002, η^2^ = 0.31	*F*2_(1, 159)_ = 40.5, *p* < 0.001, η^2^ = 0.20
Congruence × Allocutive^***^	*F*1_(1, 26)_ = 19.1, *p* < 0.001, η^2^ = 0.42	*F2*_(1, 159)_ = 15.3, *p* < 0.001, η^2^ = 0.09

*,**,*** *Indicate p-value < 0.5, 0.01, and 0.001, respectively*.

#### Acceptability judgment

Accuracy showed a main effect of Allocutive, with participants less accurate for masculine than feminine items by three percentage points, and a significant interaction between Congruence and Allocutive both by subject and by item. *Post-hoc* analysis showed that participants were less accurate for incongruent masculine items than incongruent feminine items [*t*_(26)_ = 2.85, *p* = 0.032], with a mean difference of 6.5 percentage points. All other comparisons were not significant (*p*s > 0.05). RTs showed no significant effects except for a marginally significant effect of Congruence that was only present in the analysis by item, with faster RTs to incongruent items.

#### Naturalness rating

Naturalness ratings showed main effects of Allocutive and Congruence, with participants rating masculine items more natural than feminine by 0.4 points and rating congruent items rated as more natural than incongruent by 2.2 points. There was also a significant interaction between the two factors. *Post-hoc* analysis showed that participants rated masculine incongruent items as more natural than feminine incongruent items [*t*_(26)_ = 2.45, *p* = 0.019], with a mean difference of 0.68 naturalness points. Other significant comparisons showed that incongruent feminine items were rated as less natural than congruent feminine items (by 2.5 points) and congruent masculine items (by 2.6 points), and incongruent masculine items were rated as less natural than congruent masculine items (by 1.9 points) and congruent feminine items (by 1.8 points) [all *p*s < 0.001].

### Correlations

**Figure 2:**
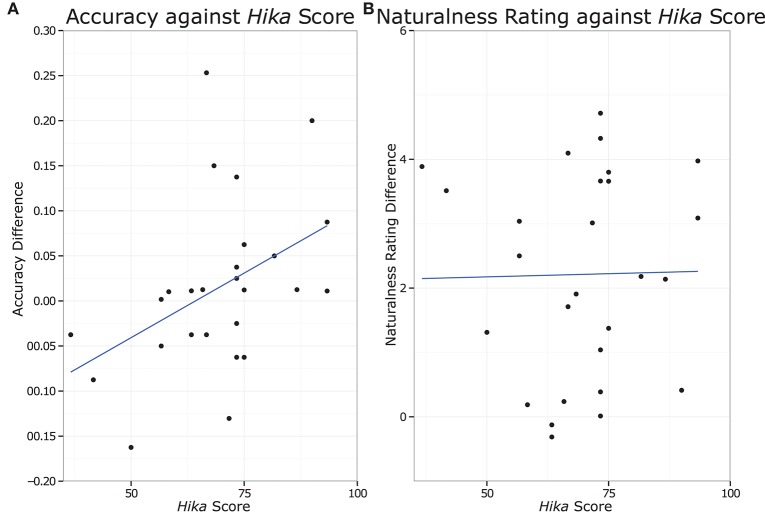
The difference in experimental measures between congruent and incongruent trials plotted against *hika* score. **(A)** Shows the difference in accuracy for the acceptability judgment. **(B)** Shows the difference in naturalness ratings.

Given the significant main effect and interaction for congruence, a Pearson's product-moment coefficient was calculated between an *hika* proficiency score and the congruence effect for accuracy and naturalness ratings. The *hika* proficiency score was calculated by averaging percent of *hika* usage during a typical week, speaking score, and comprehension score. The congruence effect was calculated by subtracting accuracy or naturalness rating for incongruent items from the results for congruent items. There was a significant correlation for accuracy [*r* = 0.43, *p* = 0.03], but not for naturalness ratings [*r* = 0.02, *p* = 0.93], as shown in Figure 2. The correlations were also computed for accuracy for only masculine items [*r* = 0.32, *p* = 0.11] and only feminine items [*r* = 0.35, *p* = 0.08], and correlations comparison showed that these two correlations were not significantly different [Pearson and Filon's *z* = 0.13, *p* = 0.90].

### Person results

**Figure 3:**
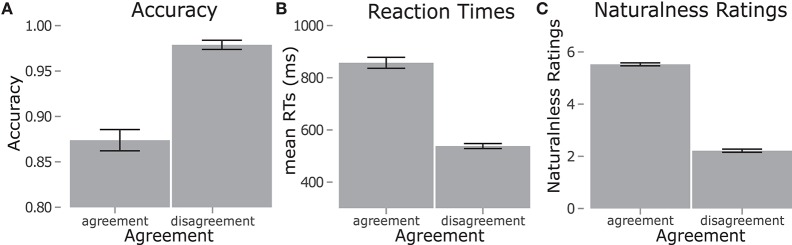
Results for the person manipulation. **(A,B)** Show accuracy and RTs respectively for the acceptability judgments. **(C)** Shows naturalness ratings results. Error bars show standard error.

The ANOVA results for the person manipulation are summarized in Table 4. Accuracy, RTs, and naturalness ratings all showed a main effect of agreement, with lower accuracy (10.5 percentage points difference), slower RTs (340 ms difference), and higher naturalness ratings (3.31 points difference) for items with person agreement. These results are shown in Figure 3. Pearson product-moment coefficients were calculated for subjects' results between tasks. RT was correlated with naturalness ratings [*r* = 0.30, *p* = 0.03], but there was no relationship for accuracy [*r* = −0.22, *p* = 0.10].

**Table 4:** ANOVA results for person manipulation conditions with by subject (F1) and by item (F2) results.

**Person Condition**		
Accuracy^***^	*F1*_(1, 26)_ = 14.3, *p* < 0.001, η^2^ = 0.35	*F2*_(1, 59)_ = 50.6, *p* < 0.001, η^2^ = 0.46
RTs^***^	*F1*_(1, 26)_ = 34.6, *p* < 0.001, η^2^ = 0.57	*F2*_(1, 59)_ = 147.4, *p* < 0.001, η^2^ = 0.71
Naturalness ratings^***^	*F1*_(1, 26)_ = 74.9, *p* < 0.001, η^2^ = 0.74	*F2*_(1, 59)_ = 1530, *p* < 0.001, η^2^ = 0.96

*** *Indicate p-value < 0.001*.

## Discussion

In this study, Basque allocutive agreement was interrogated experimentally to examine the role of addressee identity in morphosyntactic processing. This is the first experimental investigation of allocutivity and one of the first studies to consider the role of addressee identity in processing. We predicted effects for allocutive and person manipulations in both the acceptability judgment and naturalness rating tasks. The two tasks differed in the type of response and in the time constraint, and these differences may capture different stages and aspects of processing (Sorace and Keller, 2005; Marinis, 2010; Lewis and Phillips, 2015). The results met some of our hypotheses but not others, with the following significant effects: for the person manipulation, strong effects of Agreement for acceptability judgments and naturalness ratings; for the allocutive manipulation, a main effect of Congruence for naturalness ratings, a main effect of Allocutive for accuracy and naturalness ratings, and an interaction between Congruence and Allocutive for accuracy and naturalness ratings.

The effects of the person manipulation were clear. Agreement had the predicted effects for RT and naturalness ratings, with person disagreement items rejected faster and more accurately and rated as less natural. These results show that participants could detect person violations more easily than correct agreement, which is in line with other studies using acceptability judgments for person agreement (Mancini et al., 2011a,b; Perez et al., 2012; Zawiszewski et al., 2016). Results for the allocutive manipulation were not as strong, with no effect seen in RTs for the acceptability judgment task. This difference between the results for allocutive and person manipulations suggests that the underlying morphosyntactic processing may not be the same, meaning that a non-argument referent is not processed like an argument referent, despite both being represented in morphosyntax. Naturalness ratings showed effects for both manipulations, but the magnitude of the effect of Agreement was larger than that of Congruence. This shows that person violations are more disruptive to processing than allocutive incongruences for online and offline tasks. This points to a distinction between linguistic and extralinguistic information in processing, even when both are explicitly present in morphosyntax.

Although not as strong, the effects of the allocutive manipulation reveal important information about the role of addressee identity in morphosyntactic processing. Furthermore, because participants successfully completed the tasks for person manipulation, it appears that using the standard Batua dialect for the materials was not a problem and the results for the allocutive manipulation should be reliable. The main effect of Congruence shows that addressee identity was important for offline naturalness ratings (with incongruent items rated as less natural than congruent ones), but it was less relevant for online judgments. Addressee information must have been integrated during parsing for participants to detect a difference in naturalness, which means addressee identity, like speaker identity (Hanulíková et al., 2012; Hanulíková and Carreiras, 2015), impacts morphosyntactic analysis. However, a main effect of Congruence was not seen in the speeded acceptability judgment task, as was predicted based on broad evidence for online processing of speaker identity. This may mean that speaker and addressee identity do not equally impact processing; for instance, listeners may quickly extract the identity of the speaker from the speech signal, but retrieve addressee identity later via a different mechanism. These results expand the current research on the processing of addressee identity (Jiang et al., 2013; Jiang and Zhou, 2015a,b) and the relationship between pragmatic and morphosyntactic information (Momo et al., 2008; Yoshimura and MacWhinney, 2010). Given the evidence for an effect of speaker identity on morphosyntactic processing (Hanulíková et al., 2012; Hanulíková and Carreiras, 2015) and the lack of a main effect in the acceptability judgment task in the present study, it may be that the interaction between pragmatic and morphosyntactic information differs depending on the type of context information or morphosyntactic structure.

The main effect of Allocutive shows that participants were less accurate and gave higher naturalness ratings for masculine items than feminine items; this effect is best qualified by considering the interactions between Congruence and Allocutive, which suggest a more intricate addressee effect. Masculine incongruent items were more accepted and rated as more natural than feminine incongruent items, which matches our predictions from reports of higher frequency of *hika* use between men and masculine allocutive forms used for female addressees (Echeverria, 2003). This asymmetry between the two genders of allocutive verb forms further agrees with the results from our biographical questionnaire (where all possible interlocutor combinations were considered), with 85% of participants reporting that they heard more masculine than feminine allocutive forms and all speakers rating *hika* use between two male interlocutors as most typical. The usage and frequency results together with the experimental results indicate that feminine incongruent items are easier to detect and the masculine allocutive form is generalized to female addressees. It is interesting that although Basque has no other grammatical gender, there is still an asymmetry for masculine and feminine allocutive verbs. Importantly, the present behavioral study did not include conversations with two female interlocutors. This additional conversational context could conceivably produce different behavioral results from those reported here for two male or one male and one female interlocutor based (e.g., Carli, 1990). As the first experimental investigation of allocutive processing, the present study focused on the most typical social contexts where *hika* is produced and heard (i.e., with a male interlocutor). Future studies are needed to generalize the present findings to less typical conversation settings (i.e., two female interlocutors).

To explore the addressee effect more deeply, correlation coefficients were calculated for measures with a significant effect of or interaction with Congruence, namely accuracy and naturalness ratings. The correlated variables were participants' self-reported *hika* proficiency scores and the difference between congruent and incongruent items for the measure. A significant correlation was observed between *hika* proficiency score and the addressee effect for accuracy, showing that the higher a participant's *hika* proficiency, the greater the value for congruent minus incongruent accuracy. Notably, participants' score differences cross zero, with higher magnitude differences for participants with lower and higher *hika* proficiency scores. With respect to the task, this means that participants with higher proficiency were more accepting of all items than participants with lower proficiency. This could reflect two distinct groups of *hika* users: proficient users who accept incongruent items and less proficient users who reject them. This may mean that participants with higher *hika* proficiency do not follow the normative rule of allocutive agreement with addressee gender in all cases, perhaps because they hear or use more incongruent forms, such as masculine verbs for female addressees. Participants with lower *hika* proficiency may follow rules for allocutive agreement more prescriptively. This dissociation may reflect stages of experience in allocutive processing, with progression from prescriptive rule following to usage that is more frequency based, which would agree with models that highlight the impact of frequency on parsing (e.g., Mitchell et al., 1995). A potential extension to the present experiment could consider Basque speakers who are unfamiliar with *hika* using a grammar learning paradigm and compare their task performance to that of frequent *hika* users. Alternatively, participants could be recruited based on how they acquired *hika*, through explicit instruction in the classroom or from friends and family members. These follow-up studies would test directly how allocutive processing changes as a function of proficiency and type of learning. This would enable further investigation of how experience differentially modifies pragmatic and morphosyntactic processing, as well as providing insight into questions about competence and performance for *hika* usage.

The results further suggest a fundamental difference between the acceptability judgment and naturalness rating tasks. Importantly, results for the two tasks in the allocutive manipulation were not correlated across participants, showing that they capture different aspects of processing. Ideally, the RTs measured from the time-constrained acceptability judgment task capture activity during online processing (Sternberg, 1969), while the untimed naturalness ratings allowed for participants to use offline or reflective processes (Marinis, 2010). A possible explanation for our task-dependent results is that listeners only consider addressee identity when meta-linguistic, reflective processes are involved, at least for morphosyntactic analysis. A question that follows is whether all social context information is equal in processing. In light of multiple experiments showing an early effect of speaker identity (Van Berkum et al., 2008; Bornkessel-Schlesewsky et al., 2013; Hanulíková and Carreiras, 2015), the results of the present study would be consistent with listeners having earlier access to speaker than to addressee identity. However, this timing hypothesis remains to be confirmed with further studies using techniques with a fine-grained temporal resolution, such as ERPs. The critical difference between the acceptability judgment and naturalness rating might also be related to the type of question used. The acceptability judgment measured a binary, prescriptive acceptance of allocutive congruence; since participants completed this task with high accuracy, they were clearly able to make this judgment successfully. The naturalness rating, on the other hand, permitted a ranking based on actual usage, which may be more sensitive to the non-prescriptive usage of *hika*. This distinction therefore allowed us to capture different facets of the same phenomenon. Since *hika* is mainly used in speech and informal conversations, it might be easier to detect incongruences when participants provide a response on a gradient scale which is not based on grammatical results, but rather on what is heard and produced on a daily basis. A third possibility is that our behavioral measure of online processing, the acceptability judgment, is not sensitive enough to the addressee effect. This would agree with work done with the speaker effect that showed an effect in ERP analysis but absent in behavioral results (Bornkessel-Schlesewsky et al., 2013).

To conclude, this first psycholinguistic investigation of Basque allocutive agreement gives insight into the weight that extralinguistic information, namely addressee gender, has on morphosyntactic processing. The present findings have three important implications. First, addressee gender affects morphosyntactic processing for offline naturalness ratings, and this effect is modulated by the gender of the allocutive verb form. However, there was no main effect for speeded acceptability judgments, which may mean that addressee identity congruence only affects reflective, metalinguistic processing. This also suggests that addressee identity is not processed as early and automatically as is speaker identity, which would mean that different types of context information are processed differently. Second, person violations were more disruptive than allocutive incongruence, suggesting that non-argument agreement is qualitatively different from argument agreement for processing. Third, the correlations between experimental measures and biographical information showed a possible link between experience and morphosyntactic processing. Further investigation will be needed to corroborate these hypotheses, especially using a method with fine time resolution such as ERP analysis.

## Ethics statement

This study was carried out in accordance with the recommendations of BCBL Ethics Committee with written informed consent from all subjects. All subjects gave written informed consent in accordance with the Declaration of Helsinki. The protocol was approved by the BCBL Ethics Committee.

## Author contributions

SM and SC contributed equally to the design of the study. All authors were involved in either recording or editing the experimental stimuli. All authors were involved in participant recruitment and MW collected the data. All authors contributed to data analysis and interpretation. The manuscript was written by MW with input from SM and SC.

### Conflict of interest statement

The authors declare that the research was conducted in the absence of any commercial or financial relationships that could be construed as a potential conflict of interest.

## Footnotes

^1^Participant sex was originally included in the analysis, but no significant effects were observed for this factor and removing it did not change results.
